# System L Amino Acid Transporter LAT1 is required for Mouse Intestinal Epithelial Homeostasis

**DOI:** 10.7150/ijbs.128412

**Published:** 2026-04-16

**Authors:** Lingyu Bao, Zhaoyi Peng, Bingyin Shi, Yun-Bo Shi

**Affiliations:** 1 Section on Molecular Morphogenesis, Eunice Kennedy Shriver National Institute of Child Health and Human Development, National Institutes of Health, Bethesda, Maryland, MD, USA.; 2 Department of Neurology, The First Affiliated Hospital of Xi'an Jiaotong University, No.277, Yanta West Road, Xi'an, Shaanxi, 710061, P.R. China.; 3 Department of Endocrinology, The First Affiliated Hospital of Xi'an Jiaotong University, No.277, Yanta West Road, Xi'an, Shaanxi, 710061, P.R. China.

**Keywords:** amino acid transporter slc7a5, intestine, organ homeostasis, mTORC1 signaling pathway, Paneth cell, stem cell

## Abstract

The adult mammalian intestinal epithelium is constantly self-renewed via cell proliferation in the crypt. Earlier studies have revealed many genes and pathways important for regulating intestinal epithelial cell proliferation and differentiation to maintain adult epithelial homeostasis. Of interest among them is system L amino acid transporter 1 (*LAT1*), also known as slc7a5. Slc7a5 can transport thyroid hormone and large, neutral amino acids such as leucine and tryptophan. It can activate mTORC1 to increase cell proliferation by transporting amino acid. We have previously shown that slc7a5 is highly expressed in adult mouse intestinal crypt and that intestinal epithelial cell-specific knockout (^ΔIEC^) of slc7a5 (*Slc7a5*^ΔIEC^) reduces mTORC1 signaling. Unexpectedly, *slc7a5*^ΔIEC^ intestinal crypts have increased cell proliferation in the small intestine. There is also a drastic reduction in mature Paneth cells, suggesting a possible indirect effect of slc7a5 on cell proliferation by regulating secretory cell differentiation. Here, we have generated a tamoxifen-inducible intestinal epithelial-specific slc7a5 knockout line (slc7a5*^ind∆IEC^*). We show that inducible knockout of slc7a5 in adult mice also leads to reduced mature Paneth cells and increased cell proliferation in the crypt, revealing that slc7a5 is important for adult intestinal epithelial homeostasis. Kinetically, the reduction of mature Paneth cells occurs before the increase in cell proliferation. Furthermore, in stable intestinal epithelial cell-specific knockout (*Slc7a5*^ΔIEC^) animals, a reduction in mature Paneth cells occurs soon after mature Paneth cells are first formed during post-natal development while an increase in crypt cell proliferation occurs later by postnatal day 28 after intestinal maturation is complete. These findings support a mechanism where slc7a5 affects cell proliferation indirectly by regulating Paneth cell differentiation to maintain adult intestinal epithelial homeostasis.

## Introduction

The intestine has long been used as a model to study the function and properties of adult organ-specific stem cells 1-4. This is in part because of the need to repair/replace damaged intestinal epithelium due to its exposure to external harmful materials/organisms from ingested food 4-6. In mice, the intestinal epithelium, the main functional tissue of the organ, is self-renewed every 3-5 days, although different epithelial cells have different lifespans with a few months of turnover time for Paneth cells 1, 3, 7, 8. This self-renewal begins with the proliferation of transit amplifying cells that are derived from adult intestinal stem cells. As the daughter cells of transit amplifying cells migrate along the crypt-villus axis, they gradually differentiate into different types of epithelial cells, eventually dying off via apoptosis mainly nearly the tip of the villus. Many studies have revealed the involvement of different signaling pathways, such as Wnt and BMP pathways, during intestinal development and homeostasis 1, 3, 4, 9.

We have previously shown that knocking out system L amino acid transporter 1 (LAT1), also known as slc7a5, leads to adult intestinal defects in mice 10. Slc7a5 forms a heterodimer with the heavy chain CD98 (slc3a2), which is necessary for membrane localization and transporter function of slc7a5. The full length slc7a5 was first identified as a thyroid hormone-induced gene (IU12) during *Xenopus* metamorphosis and subsequently shown to encode the transporter subunit of the slc7a5/slc3a2 heterodimer to transport both amino acids and thyroid hormone 11-16. Slc7a5 has been implicated to be important for immune system, placenta, blood brain barrier, and protecting the body against pathogens 17, 18. Consistent with a critical role of the transporter in development and/or organ function, global knockout of slc7a5 or disruption of transporter function by altering the extracellular domain of CD98 leads to embryonic lethality in mice 19, 20. In addition, conditional knockout of slc7a5 leads to defects in different tissues/organs including the intestine, skeletal muscle, T cells, osteoclasts, and hypothalamic neurons, consistent with the broad expression patterns of the transporter 10, 21-24. These defects caused by the slc7a5 knockout have often been linked to alterations in mTORC1 signaling associated with the amino acid transporter function of slc7a5.

In the adult mouse intestine, epithelial-specific knockout of slc7a5 leads to elongated crypts and increased cell proliferation, unexpected based on the reduced mTORC1 activity caused by the knockout 10. There is also a dramatic decrease in mature Paneth cells in the crypts while goblet cells are decreased in the villi but increased in the crypts of the knockout intestine. Cell death in the epithelium is increased by the knockout, leading to faster epithelial cell turnover in the adult to maintain epithelial homeostasis. To determine whether these knockout effects are caused by altered intestinal development and/or adult homeostasis, we generated tamoxifen-inducible intestinal epithelial-specific slc7a5 knockout mice (slc7a5*^ind∆IEC^*). We found that inducible knockout of slc7a5 in adult mice also led to the same phenotypes as the stable epithelial-specific slc7a5 knockout. Interestingly, kinetic analyses revealed that the reduction in mature Paneth cells occurred prior to the increased cell proliferation. In addition, stable epithelial-specific slc7a5 knockout neonates had reduced mature Paneth cells by the weaning stage (postnatal day 21) while an increase in crypt cell proliferation occurred later at postnatal day 28. Our findings suggest that slc7a5 is critical for maintaining Paneth cell differentiation, which may in turn help maintain adult intestinal epithelial homeostasis.

## Results

### Slc7a5 is required for maintaining Paneth cell differentiation in adult mice

**Figure 1:**
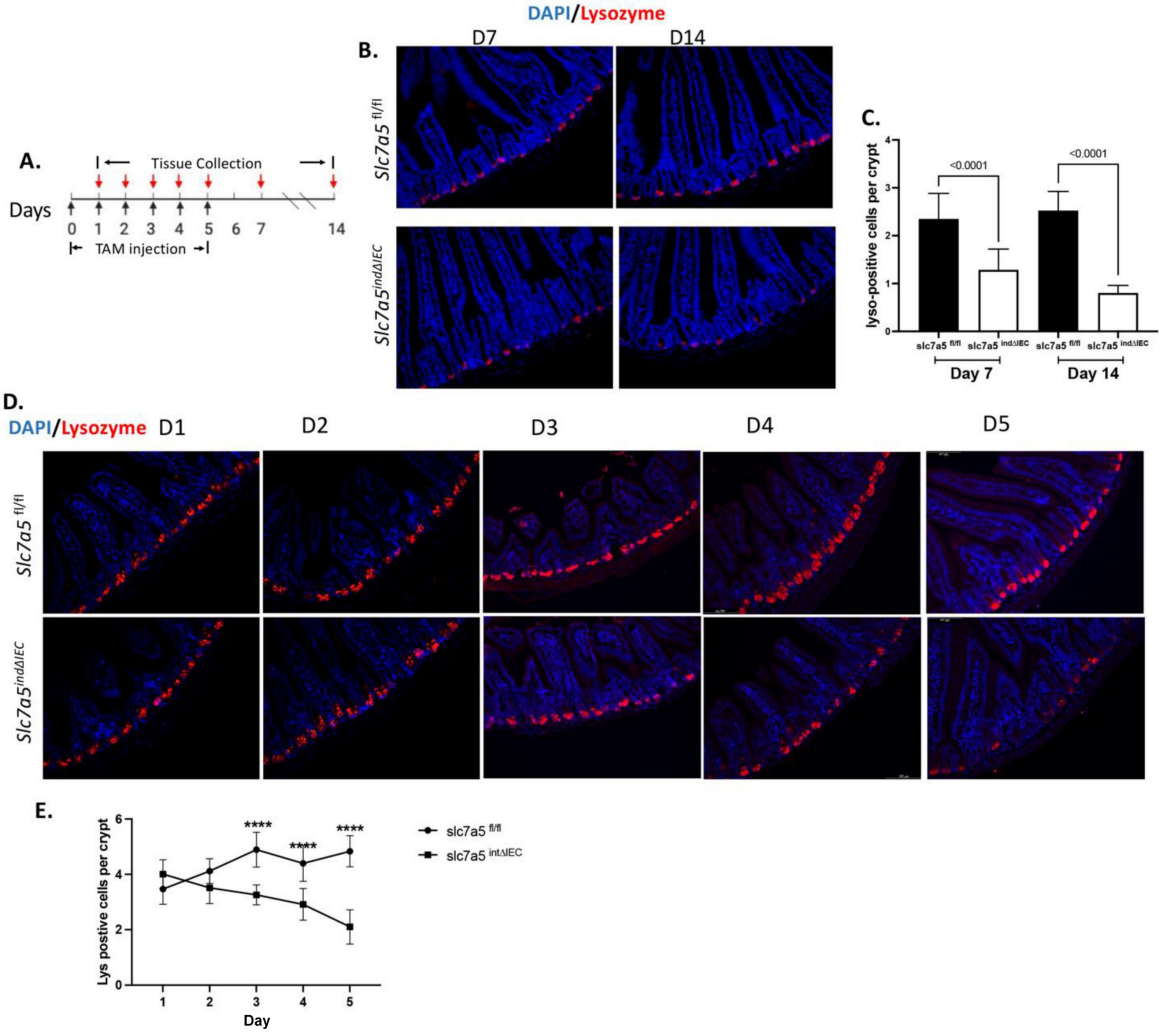
** Inducible knockout of slc7a5 in adult intestinal epithelium leads to reduced Paneth cells. A.** Schematic diagram of inducible knockout of slc7a5 in adult intestinal epithelium with tamoxifen (TM) treatment. The slc7a5*^fl/fl^* and slc7a5*^ind∆IEC^* mice were injected with 75mg/kg TM for 1 to 5 consecutive days (D0-D4). A black arrow indicates a day when TM was given. A red arrow indicates a day when intestinal tissues were collected. **B/C.** Lysozyme-positive Paneth cells are reduced by TM treatment. Cross-sections of the small intestine were stained with DAPI (blue) for DNA and anti-lysozyme antibody (red) for Paneth cells at D7 and D14 after TM injection (B). The lysozyme-positive Paneth cells in the slc7a5*^fl/fl^* and slc7a5*^ind∆IEC^* intestine were quantified from the cross-sections, showing less lysozyme-positive cells in the crypt of *slc7a5^ind∆IEC^* mice at both treatment days. **D/E.** The reduction in Paneth cells occurs as early as 3 days after initiating TM treatment. Cross-sections of the small intestine were stained with DAPI (blue) for DNA and anti-lysozyme antibody (red) for Paneth cells at D1 to D5 after TM injection (D). The lysozyme-positive Paneth cells in the slc7a5*^fl/fl^* and slc7a5*^ind∆IEC^* intestine were quantified from the cross-sections from D1-D5 (E), revealing that the number of lysozyme-positive cells in the crypt of *slc7a5 ^ind∆IEC^* mice were significantly reduced by D3.

To investigate the role of slc7a5 in adult intestinal epithelial homeostasis, we generated an inducible epithelial-specific slc7a5 knockout mouse line by crossing homozygous slc7a5-floxed mice (slc7a5*^fl/fl^*) 10, 22 with transgenic mice expressing the inducible Cre recombinase under the control of the villin promoter (Vil-CreERT2) (Fig. 1A), which enables effective knockout of the floxed gene in the intestinal epithelium in the presence of tamoxifen 25, 26. Upon injection of tamoxifen into the resulting mice (slc7a5*^ind∆IEC^*), slc7a5 is specifically knocked out in the epithelium, while normal wild type slc7a5 continues to be expressed in slc7a5*^ind∆IEC^* mice in the absence of tamoxifen treatment or in slc7a5*^fl/fl^* mice with or without tamoxifen treatment. When adult slc7a5*^fl/fl^* and slc7a5*^ind∆IEC^* mice were treated with tamoxifen with a daily injection from day 0 to day 4 and the intestines were isolated on day 7 and day 14 for lysozyme-staining to detect mature Paneth cells, we observed that a significant reduction in mature Paneth cells in slc7a5*^ind∆IEC^* mice compared to slc7a5*^fl/fl^* mice (Fig. 1B-C). Kinetically, the reduction in mature Paneth cells was detected as early as day 3 during tamoxifen treatment (Fig. 1D-E). We have previously shown that the total Paneth cell number changes little upon stable epithelial knockout of slc7a5 even though mature Paneth cell number decreases dramatically 10, just like that we observed here. This and the fact that Paneth cells have a lifespan of a few months indicate that slc7a5 is required for maintaining the mature stage of the Paneth cells and that its knockout leads to their dedifferentiation.

### The increases in crypt cell proliferation caused by epithelial slc7a5 knockout occurs after the loss of mature Paneth cells

**Figure 2:**
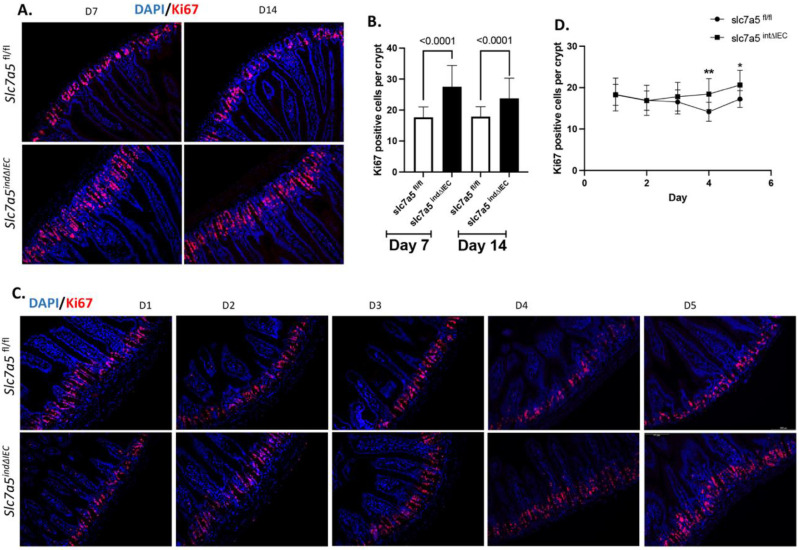
** Inducible knockout of slc7a5 in adult intestinal epithelium increases cell proliferation in the crypt. A/B.** Cell proliferation is increased by TM treatment. Cross-sections of the small intestine were stained with DAPI (blue) for DNA and anti-ki67 antibody (red) for proliferating cells at D7 and D14 after TM injection (A) and ki67-positive cells were quantified from the cross-sections, showing more ki67 positive cells in the crypt of *slc7a5 ^ind∆IEC^* mice at both days. **C/D.** Cell proliferation is increased after 4 days of TM treatment. Cross-sections of the small intestine were stained with DAPI (blue) for DNA and anti-ki67 antibody (red) for proliferating cells from D1-D5 after TM injection (C) and ki67-cells were quantified from the cross-sections from D1-D5 (D), revealing the number of the ki67 positive cells in the crypt between slc7a5*^fl/fl^* and slc7a5*^ind∆IEC^* mice starts to show significant difference at D4.

Stable intestinal epithelial knockout of slc7a5 increases crypt cell proliferation in adult mice 10. To determine if this is due to a requirement of slc7a5 to maintain normal cell proliferation in the adult intestine, we analyzed cell proliferation in the intestine of slc7a5*^fl/fl^* and slc7a5*^ind∆IEC^* mice at different days after initiating tamoxifen treatment at day 0. As shown in Fig. 2A/B, immunofluorescence staining with anti-ki67 antibody for proliferating cells at day 7 and day 14 revealed increased cell proliferating cells in the crypts of *slc7a5^ind∆IEC^* mice at both time points, indicating the slc7a5 is essential for maintaining both normal cell proliferation in the adult mouse intestine.

The observation that epithelial cell knockout of slc7a5 leads to the expected reduction in mTORC1 signaling, but an unexpected increase in cell proliferation in the crypt raises the possiblity that the loss of mature Paneth cells may indirectly cause increased cell proliferation in the crypt 10. Such a hypothesis is consistent with the observation that calorie restriction leads to inhibition of mTORC1 pathway specifically in Paneth cells, which in turn increases crypt cell proliferation by activating SIRT1 27-29. If slc7a5 affects cell proliferation indirectly via regulating Paneth cells, the loss of mature Paneth cells should precede the increase in cell proliferation. To test this, we analyzed cell proliferation in the intestine of slc7a5*^fl/fl^* and slc7a5*^ind∆IEC^* mice at earlier time points following the initiation of tamoxifen treatment at day 0. We did not observe any significant increase in cell proliferation until 4 days after the initiation of tamoxifen treatment (Day 4, Fig. 2C-D), which occurred 1 day later than the observed reduction in mature Paneth cells (Day 3, Fig. 1 D-E). Thus, slc7a5 knockout in the intestinal epithelium causes dedifferentiation of mature Paneth cells prior to any increase in crypt cell proliferation.

### Mature Paneth cells are reduced in stable intestinal epithelial-specific slc7a5 knockout neonates compared to wild type animals soon after their formation during postnatal development

**Figure 3:**
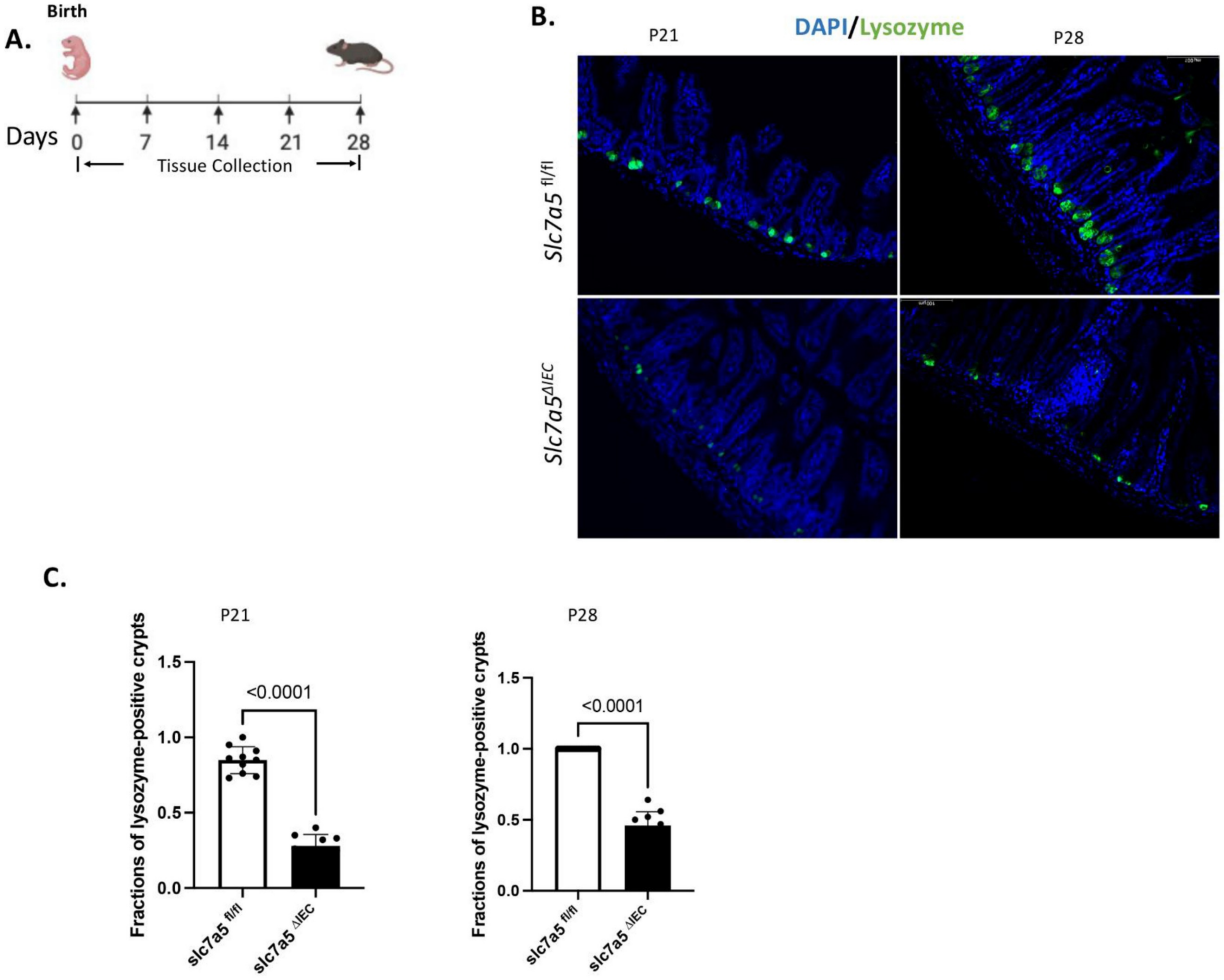
** Intestinal epithelial knockout of *slc7a5* leads to reduced Paneth cells by weaning stage. A.** Schematic diagram for the analyses of neonatal mice. Black arrows indicated the days after birth (postnatal days) when the intestine was collected. **B/C.** Cross-sections of the small intestine were stained with DAPI (blue) for DNA and anti-lysozyme antibody (green) for Paneth cells at postnatal day 21 (p21) and 28 (p28) (B) and the fraction of lysozyme-positive crypts were quantified (C, with 1 indicating all crypts having at least one lysozyme-positive Paneth cell), showing a significant reduction in the slc7a5*^∆IEC^* mice compared to the wild type (slc7a5*^fl/fl^*) mice at both P21 and P28 (note that there are few mature Paneth cells up to P14 even in wild type animals, making it difficult to quantify any differences between wild type and knockout animals on P14 or earlier).

The mouse intestine lacks any crypts at birth and intestinal maturation occurs before weaning, during the first 2-3 weeks after birth when crypts and mature Paneth cells are formed 1, 2, 30, 31. To study the potential role of slc7a5 in Paneth cell development, we generated stable intestinal epithelial-specific slc7a5 knockout mice (slc7a5*^ΔIEC^*) by crossing slc7a5*^fl/fl^* mice with transgenic mice expression Cre under the villin promoter 10. As there were few mature Paneth cells up to postnatal day 14 (P14), we analyzed mature Paneth cells by lysozyme-staining of intestinal cross-sections from wild type (slc7a5*^fl/fl^*) and knockout (slc7a5*^ΔIEC^*) mice on P21 (weaning stage) and P28 (Fig. 3). The results showed that there were drastically reduced mature Paneth cells in the knockout mice compared to the wild type animals at both time points (Fig. 3B-C). Thus, slc7a5 is also required for the formation and/or maintaining the differentiation state of mature Paneth cells during postnatal intestinal maturation.

### Stable intestinal epithelial-specific slc7a5 knockout increases cell proliferation only in mature but not developing crypts

**Figure 4:**
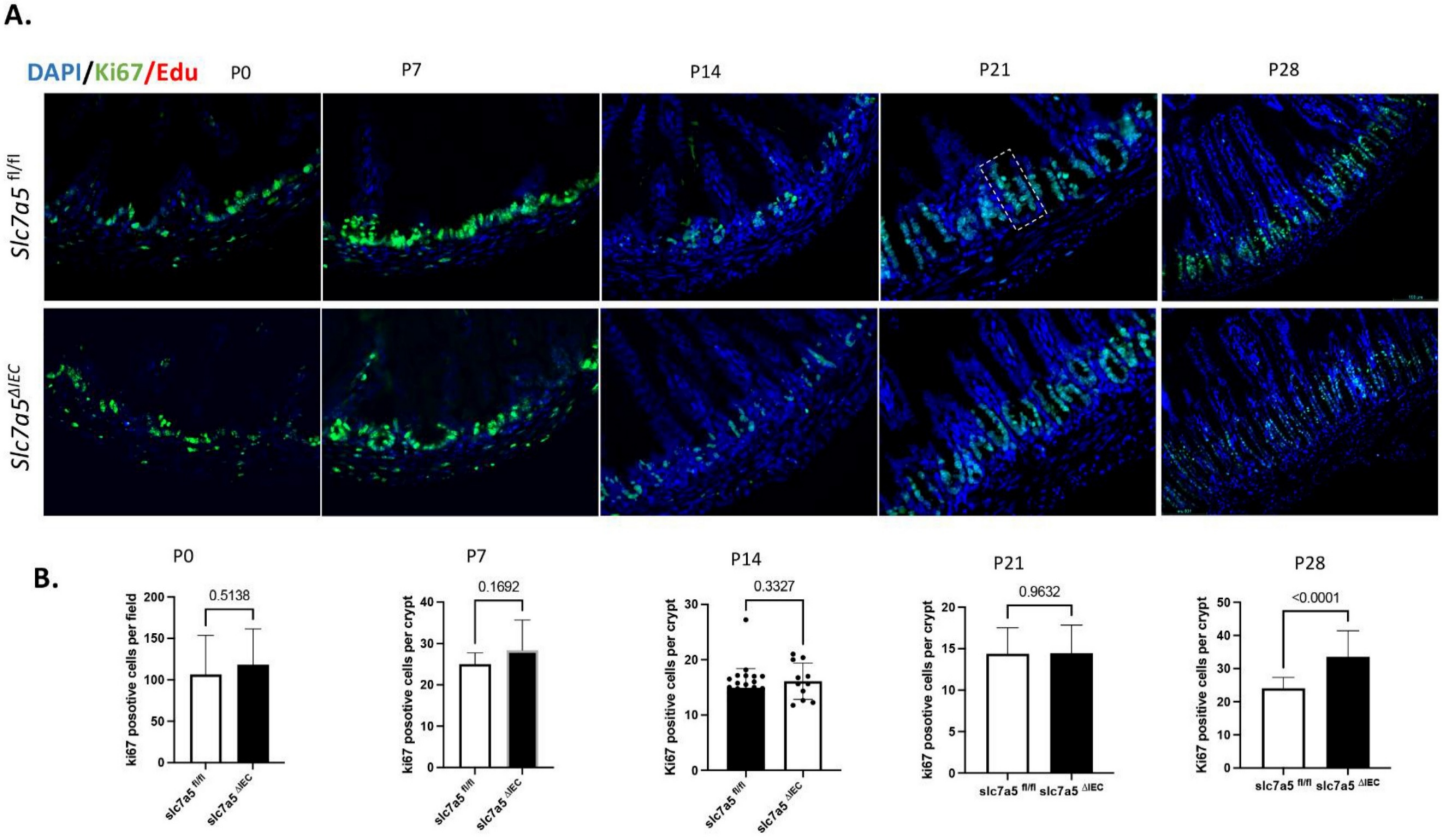
** Intestinal epithelial knockout of *slc7a5* leads to increased cell proliferation by the end of weaning stage. A/B.** Cross-sections of the neonatal small intestine were stained with DAPI (blue) for DNA and anti-ki67 antibody (green) for proliferating cells from P0 to P28 (A) and ki67-positive cells were quantified from the cross-sections (B), revealing a significant increase in cell proliferation in slc7a5*^∆^*^IEC^ mice compared to the wild type at P28 but not at P0-P21. Notes that during early postnatal stages (P0, P7, and P14) when crypts are not yet fully formed, Ki67-positive cells were quantified per field to reflect proliferative activity within the intervillus epithelial pockets. After crypt maturation (P21 and P28), Ki67-positive cells were quantified per crypt. The dashed box marks the boundary of a crypt at P21 in (A).

During postnatal intestinal maturation, crypts are formed via invasion of the cells in the inter-villus pocket into the underlying connective tissue 1, 2, 30, 31. These cells proliferate and eventually differentiate to establish the crypt-villus structure of the adult intestine. To determine if slc7a5 is required for this process, particularly the proliferation of the cells in the developing crypts, we analyzed cell proliferation by staining intestinal cross-sections from wild type (slc7a5*^fl/fl^*) and epithelial-specific knockout (slc7a5*^ΔIEC^*) mice at postnatal day 0 to day 28 when the adult intestine is formed. The results revealed that no significant changes in cell proliferation in the developing crypt up to the end of weaning (P21) (Fig. 4). By P28, the adult intestinal maturation is complete and cell proliferation in the crypts of the knockout mice was significantly increased compared to the wild type mice (Fig. 4), similar to the findings in the inducible knockout adult mice or in older slc7a5*^ΔIEC^* mice as we reported earlier 10. Furthermore, analyses of intestinal cross-sections at different time points showed no significant difference in crypt morphology up to the weaning stage. Thus, slc7a5 is not required for the development of the adult small intestinal epithelium, and its effect on crypt cell proliferation during this process also lags behind that on mature Paneth cells.

### Knockout of slc7a5 also increases cell proliferation in the colon

**Figure 5:**
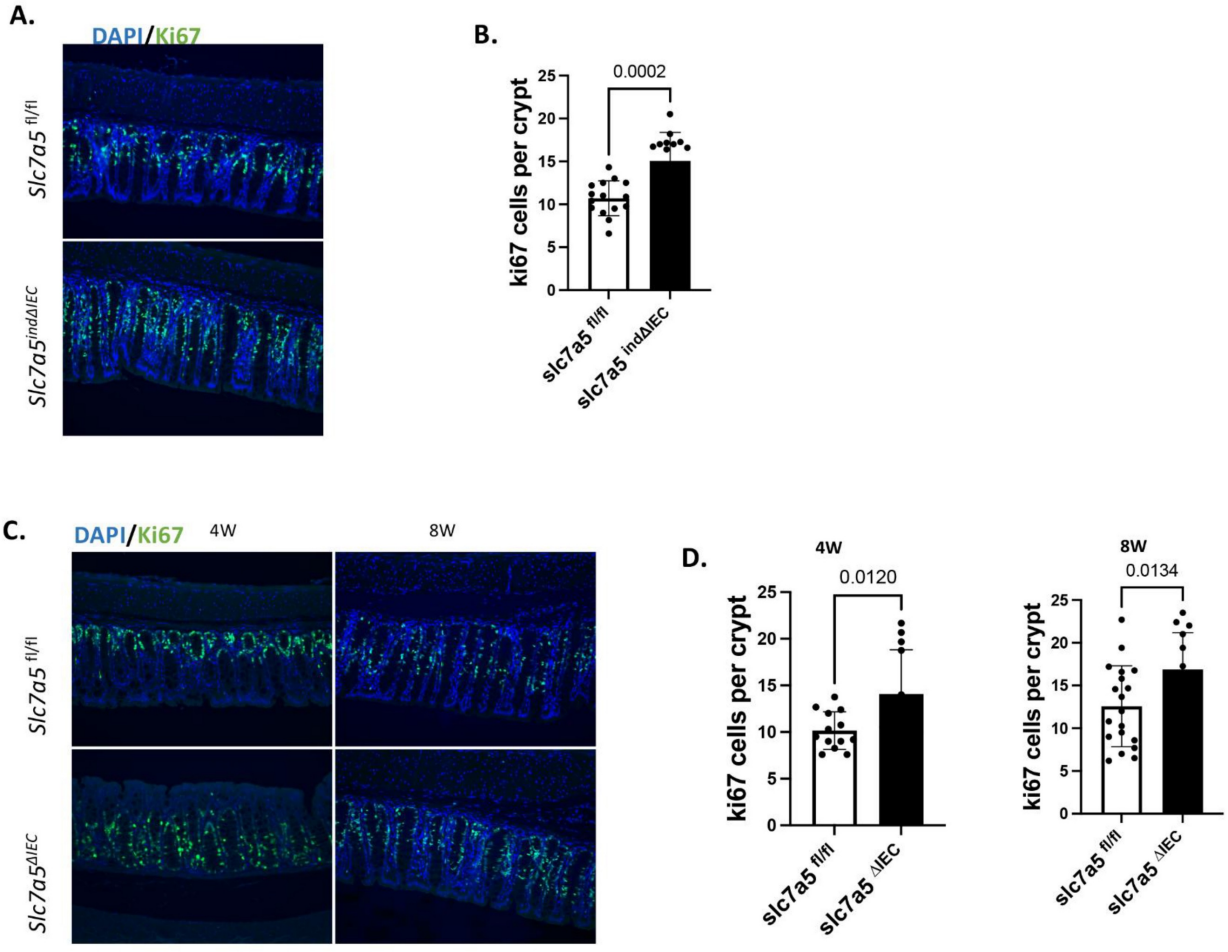
** Both stable and inducible intestinal epithelial knockout of *slc7a5* lead to increased cell proliferation in adult colon. A/B.** Cross-sections of the large intestine from slc7a5*^fl/fl^* and slc7a5*^ind∆IEC^* mice 14 days after starting TM injection as in Fig. 1 were stained with DAPI (blue) for DNA and anti-ki67 antibody (green) for proliferating cells (A) and ki67-positive cells were quantified (B), showing significantly more ki67-positive cells in slc7a5*^ind∆IEC^* large intestine. **C/D.** Cross-sections of the large intestine from 4 weeks (4W) and 8W old slc7a5*^fl/fl^* and slc7a5*^∆IEC^* (stable knockout) mice were stained with DAPI (blue) for DNA and anti-ki67 antibody (green) for proliferating cells (C) and ki67-positive cells were quantified (D), revealing significantly more ki67-positive cells in slc7a5*^∆IEC^* large intestine at both 4 weeks and 8 weeks of age.

Our findings above suggest that slc7a5 likely affects cell proliferation in the small intestine by regulating the maintenance of mature Paneth cells. As Paneth cells are absent in the large intestine (colon), we wonder if slc7a5 knockout influences cell proliferation in the colonic crypts. We stained proliferating cells by using an anti-ki67 antibody on colonic cross-sections from slc7a5*^fl/fl^* and slc7a5*^ind∆IEC^* mice 14 days after initiating tamoxifen injection and observed a significant increase in cell proliferation in the knockout colon (Fig. 5A-B). Similarly, stable epithelial knockout of slc7a5 (slc7a*^∆IEC^*) also led to increased colonic crypt cell proliferation in the mice of 4 or 8 weeks of age when compared to age-matched wild type (slc7a5*^fl/fl^*) mice (Fig. 5C-D), just like what we observed in the small intestine. Thus, slc7a5 is important for maintain proper cell proliferation in the crypts of both the small and large intestine.

## Discussion

Slc7a5 or LAT1 encodes a transporter for amino acid and thyroid hormone. The full length slc7a5 was originally identified as a thyroid hormone-induced gene during *Xenopus* metamorphosis and implicated to promote the formation and/or proliferation of adult intestinal stem cells by functioning to enhance cellular uptake of thyroid hormone during thyroid hormone-dependent metamorphosis 11, 32. As an amino acid transporter, it is believed to enhance cell proliferation by activating mTORC1 signaling. Thus, it is surprising that intestinal epithelial-specific knockout of slc7a5 leads to increased crypt cell proliferation in the small intestine of adult mice 10. Since the vertebrate intestine is constantly self-renewed throughout adult life, the observed phenotype of slc7a5 knockout mice can be due to a role of slc7a5 during intestinal development and/or intestinal homeostasis in the adult. Our studies here demonstrate that slc7a5 is required for the normal homeostasis of adult small intestinal epithelium by ensuring the maintenance of the mature state of Paneth cells and preventing excess proliferation in the crypts of small intestine. We further show that slc7a5 is not required for the development of the crypt or the formation and proliferation of adult stem cells during postnatal small intestinal maturation. It is, however, required for the formation and/or maintenance of mature Paneth cells during postnatal intestinal maturation when mature Paneth cells are first formed during development.

By using a tamoxifen-inducible Cre controlled by a transgenic villin promoter to knock out slc7a5 in the intestinal epithelium, we showed that inducible knockout of slc7a5 in adult mice also caused a reduction in mature Paneth cells and increased cell proliferation in the small intestinal crypts of adult mice, just like what we observed in adult mice with a stable epithelial-specific knockout of slc7a5 10. Thus, the intestinal phenotypes in slc7a5 knockout mice are due to an important function of slc7a5 in the maintenance of small intestinal epithelial homeostasis in the adult. Interestingly, kinetic studies by using the inducible knockout adult mouse model showed that the reduction in mature Paneth cells occurred before any significantly change in cell proliferation. Similarly, when we analyzed stable epithelial-specific slc7a5 knockout animals during postnatal intestinal maturation, we did not observe any increased cell proliferation up to postnatal day 21, the weaning stage when intestinal maturation is complete, but did observe increased cell proliferation by postnatal day 28 (4 weeks old), well after the significant reduction in mature Paneth cells detected by as early as postnatal day 21, shortly after their formation around 7-10 days after birth 31. Thus, in the small intestine, slc7a5 knockout causes the reduction in mature Paneth cells first and only subsequently, the increase in crypt cell proliferation. Give the months-long life span of Paneth cells, slc7a5 is likely required to maintain the mature state of Paneth cells. Furthermore, our previously analyses of epithelial slc7a5 knockout intestine by using single cell RNA-sequencing and electron microscope have shown that slc7a5 knockout causes Paneth cells to dedifferentiate and lose/reduce lysozyme proteins 10. As Paneth cells are important for the adult stem cell niche and a loss of Paneth cells has been associated with increase cell proliferation in the small intestinal crypts 4, 8, 9, 27-29, our findings strongly suggest that slc7a5 knockout causes Paneth cell dedifferentiation, which in turn indirectly causes increased cell proliferation possibly by altering the stem cell niche, cell-cell interactions via secreted signaling factors, and/or remodeling the extracellular matrix.

In addition, slc7a5 knockout also causes increased in cell proliferation in the large intestine or colon. Interestingly, there are no villi or Paneth cells in the colon. This makes it difficult to use temporal analyses determine if the increased cell proliferation follows any significant changes in the colon. Thus, it is unclear how slc7a5 affects cell proliferation in the colonic crypts. Nonetheless, our findings here suggest that slc7a5 influences intestinal epithelium through at least two distinct mechanisms: a Paneth-dependent indirect mechanism in the small intestine and a Paneth-independent mechanism in the colon. Further studies are needed to understand the mechanisms by which slc7a5 controls Paneth cell maturation and regulates intestinal crypt cell proliferation in both the small and large intestine. Of particular interest is the established role of slc7a5 in amino acid-dependent mTORC1 signaling. Genetic alterations of mTORC1 activity, especially in the Paneth cells, or pharmacological modulations of mTORC1 signaling with pathway agonists or inhibitors in intestinal organoids of wild type or slc7a5 knockout mice may help determine whether altered mTORC1 activity contributes causally to the increased proliferation observed in slc7a5-deficient intestine.

Finally, earlier studies have suggested that slc7a5 is important for the formation and/or proliferation of the adult intestinal stem cells during* Xenopus* metamorphosis by functioning as a thyroid hormone transporter 11, 13-15. This and the conservation in intestinal maturation among vertebrates 2 suggest that slc7a5 knockout may affect the formation and/or proliferation of the adult stem cell during postnatal mouse intestinal maturation. Surprisingly, we failed to detect any defects in crypt morphology or cell proliferation in the crypts during the first 3 weeks after birth when mouse small intestine matures. Thus, slc7a5 is not required for the formation of the crypt and adult stem cells, nor for their subsequent proliferation during postnatal development. It is possible that slc7a5 is not involved in these processes in mammals. Alternatively, the role of slc7a5 may be substituted/compensated by other transporters in the knockout mice. Future analyses of other transporters with similar substrate specificities should help to resolve this.

## Materials and Methods

### Animals

Slc7a5*^fl/fl^* mice were previously described 22. Villin-Cre mice (The Jackson Laboratory) and tamoxifen-inducible Cre under the same promoter (Vil-CreERT2) (The Jackson Laboratory), backcrossed to C57BL/6J mice for more than 10 generations, were bred with Slc7a5*^fl/fl^* mice to produce slc7a5*^ΔIEC^* and slc7a5*^ind∆IEC^* mice for stable and inducible epithelial knockout of Slc7a5, respectively. Slc7a5*^fl/fl^* mice were used as the wild type controls for both slc7a5*^ΔIEC^* and slc7a5*^ind∆IEC^* mice. The slc7a5*^ΔIEC^*, slc7a5*^ind∆IEC^*, and slc7a5*^fl/fl^* mice were sex- and age- matched littermates and cohoused. For induction of slc7a5 knockout in the CreERT2 line (slc7a5fl/fl; vil-CreERT2, slc7a5*^ind∆IEC^*), tamoxifen (Sigma-Aldrich) was emulsified in corn oil at a concentration of 20 mg/ml and injected daily intraperitoneally at 100 μl per adult mouse (8-10 weeks old) for 1-5 consecutive days and euthanized at the indicated time points after first tamoxifen injection (Fig. 1A). For neonatal study, slc7a5*^fl/fl^* mice and slc7a5*^ΔIEC^* lines were used, and the animals euthanized at indicated postnatal day (Fig. 3A). All mice were maintained in accordance with the NIH animal facility guidelines for laboratory animal research. All animal care and treatments were done as approved by Animal Use and Care Committee of Eunice Kennedy Shriver National Institute of Child Health and Human Development, National Institutes of Health.

### Immunohistochemistry

The intestine was removed from age-matched slc7a5*^fl/f^*, slc7a5*^ΔIEC^*, or slc7a5*^ind∆IEC^* littermates, flushed with ice-cold 1xPBS buffer, and fixed in 4% formaldehyde (and if needed, stored at 4 °C), followed by embedding in paraffin and then cutting to 5 µm sections. For H&E staining, the 5 µm sections were stained with hematoxylin and eosin following manufacturer's protocol (Sigma) and analyzed under a bright field microscope. For immunofluorescent staining, the 4-5 μm paraffin sections/animal were deparaffinized in xylene and rehydrated in a series of different concentrations of ethanol. Antigen retrieval was performed by boiling in an antigen retrieval buffer (1 mM Tris, 1 mM EDTA and 0.05% Tween-20) for 3 min at 125 °C followed by washing the slides under running water and rinsing them in 1xTBS-Tween (Tris buffered saline plus 0.1% tween-20) for 5 min. After incubation in blocking buffer (10% normal goat serum in PBS) for 1 h at room temperature, the primary antibody was added, and the slides were incubated at 4 °C overnight. The slides were then washed in 1xTBS-Tween and subsequently incubated with a fluorescence-labeled secondary antibody for 1 h at room temperature. Next, the slides were washed three times with 1x TBS-Tween and covered with DAPI-containing mounting medium to counterstain the DNA. The fluorescent pictures for different colors and/or different sections were taken under the same settings. The fluorescent pictures were analyzed by using the Image J at the same setting to count positive cells. The primary antibodies used were anti-lysozyme antibody (Abcam, and Dako) and anti-Ki67 antibody (Cell Signaling Technology).

### Statistical analysis

Representative images of at least two independent experiments with each having at least three mice per sample group were shown. All statistical analyses and graphs were performed/generated by using GraphPad Prism version 8.0 (GraphPad Software, La Jolla, CA). Student's t test was used to examine the differences between groups and a P < 0.05 was considered statistically significant. For histological analysis, multiple sections from each animal were used and 10 to 45 sections in total were analyzed for each group. All data were expressed as mean ± SD.
